# Endotoxin-free purification for the isolation of Bovine Viral Diarrhoea Virus E2 protein from insoluble inclusion body aggregates

**DOI:** 10.1186/1475-2859-10-57

**Published:** 2011-07-26

**Authors:** Antonino S Cavallaro, Donna Mahony, Margaret Commins, Timothy J Mahony, Neena Mitter

**Affiliations:** 1 Queensland Agricultural Biotechnology Facility, Agri-Science Queensland, Queensland, Australia; 2 Queensland Alliance for Agriculture and Food Innovation, The University of Queensland, Queensland, Australia; 3 Queensland Agricultural Biotechnology Centre, The University of Queensland, St Lucia, Queensland, 4072 Australia

## Abstract

**Background:**

Protein expression in *Escherichia coli *may result in the recombinant protein being expressed as insoluble inclusion bodies. In addition, proteins purified from *E. coli *contain endotoxins which need to be removed for *in vivo *applications. The structural protein, E2, from Bovine Viral Diarrhoea Virus (BVDV) is a major immunogenic determinant, and is an ideal candidate as a subunit vaccine. The E2 protein contains 17 cysteine residues creating difficulties in *E. coli *expression. In this report we outline a procedure for successfully producing soluble and endotoxin-free BVDV E2 protein from inclusion bodies (IB).

**Results:**

The expression of a truncated form of BVDV-E2 protein (E2-T1) in *E. coli *resulted in predominantly aggregated insoluble IB. Solubilisation of E2-T1 with high purity and stability from IB aggregates was achieved using a strong reducing buffer containing 100 mM Dithiothreitol. Refolding by dialysis into 50 mM Tris (pH 7.0) containing 0.2% Igepal CA630 resulted in a soluble but aggregated protein solution. The novel application of a two-phase extraction of inclusion body preparations with Triton X-114 reduced endotoxin in solubilised E2-T1 to levels suitable for *in vivo *use without affecting protein yields. Dynamic light scattering analyses showed 37.5% of the protein was monomeric, the remaining comprised of soluble aggregates. Mice immunised with E2-T1 developed a high titre antibody response by ELISA. Western hybridisation analysis showed E2-T1 was recognised by sera from immunised mice and also by several BVDV-E2 polyclonal and monoclonal antibodies.

**Conclusion:**

We have developed a procedure using *E. coli *to produce soluble E2-T1 protein from IB, and due to their insoluble nature we utilised a novel approach using Triton X-114 to efficiently remove endotoxin. The resultant protein is immunogenic and detectable by BVDV-E2 specific antibodies indicating its usefulness for diagnostic applications and as a subunit vaccine. The optimised *E. coli *expression system for E2-T1 combined with methodologies for solubilisation, refolding and integrated endotoxin removal presented in this study should prove useful for other vaccine applications.

## Background

Bovine viral diarrhoea virus (BVDV) infection of cattle is linked to economically important diseases with losses in the USA being estimated to US$10-40 million per million calves [1] and US$6 million per million calves in the UK [2]. BVDV is a member of the Pestivirus genus within the Flavivirus family. The BVDV genome is a positive sense RNA molecule with one open reading frame (ORF) encoding for a polyprotein which is cleaved into the structural and non-structural proteins [3]. Of the structural proteins, the surface glycoprotein, E2 is a major immunogenic determinant and is involved in virus neutralisation [4]. E2 is therefore an ideal candidate for use in subunit vaccines [5,6].

E2 contains 17 cysteine residues which form both intramolecular disulphide bonds and intermolecular disulphide bonds resulting in dimers of E2-E2 and E2-E1 [7]. For a protein with high disulphide bond formation, recombinant protein expression is best attempted in mammalian and insect cell systems [8]. Expression of E2 has been documented in mammalian [5,9] as well as insect cell lines [5,10,11] and an insect larval system [12]. Mammalian and insect cell line expression of proteins have the advantage of producing proteins with correct conformation and post-translational modifications such as glycosylation, but generally yields are lower than *Escherichia coli *systems. However, the low costs required for veterinary vaccine applications preclude the use of more expensive protein expression systems such as mammalian and insect cell systems [13]. The use of *E. coli *based expression systems is hindered by the fact that although the recombinant proteins are generally expressed at high yields the resulting proteins are often insoluble and lack post-translational modifications [8]. Expression of glycosylated proteins in *E. coli *has been previously reported, Chia et al. [14] demonstrated the successful generation of neutralising antibodies to the envelope protein (E) of Japanese Encephalitis Virus (JEV), also a member of the Flavivirus family. Das et al. [15] demonstrated monoclonal antibodies generated against *E. coli *expressed Ebola virus antigen recognised the glycosylated antigen expressed in mammalian cells.

Expression of recombinant proteins in *E. coli *often leads to insoluble aggregates known as IB [16]. Though, usually seen as an undesirable effect, recent research has shed light on advantages of IB formation [17]. Furthermore as IB aggregates are observed due to intermolecular interactions among a single type of protein, the formation of IB can aid in the purification and isolation of the expressed protein [18]. IB aggregates are common in proteins containing disulphide bonds (such as E2), as the reducing environment of the bacterial cytosol inhibits the formation of disulphide bonds [8,19]. IB solubilisation is generally achieved by the use of chaotropic agents, such as urea and guanidine salts, and/or detergents and reducing agents. Refolding of the protein can be achieved by dilution or dialysis into suitable buffers that may contain detergents, oxidising or reducing agents and other additives to maintain solubility and to facilitate correct folding of the protein [19,20].

A potential drawback from the production of recombinant proteins in *E. coli *is contamination with endotoxins. Endotoxins are a major component of gram negative bacterial cell walls and are liberated during extraction of proteins [21]. Endotoxins are heat stable lipopolysaccharides with monomer molecular weights ranging from 10 to 20 kDa and form highly stable aggregates [21]. Mammalian exposure to endotoxins can induce several undesirable physiological effects such as fever, leukocytosis, hypoferremia, platelet aggregation, thrombocytopenia and coagulapathies [22]. Common methods employed for the removal of endotoxins from protein purifications include ultrafiltration, adsorption techniques, affinity chromatography and Triton X-114 two-phase extraction [21,23]. Two-phase extraction with Triton X-114 showed 98-99% reduction of endotoxin levels for the soluble proteins cardiac troponin I, myoglobin and creatin kinase with a protein recovery of > 90% [24]. Aida and Pabst [25] demonstrated a 1000 fold reduction of endotoxin for the soluble proteins catalase, cytochrome c, and bovine serum albumin using this method with a protein loss of 2% for cytochrome c.

In this study, we report the expression and purification of a truncated form of the BVDV E2 protein (E2-T1) from IB aggregates and optimised solubilisation conditions for this protein. We also describe a novel process for the removal of endotoxins from IB aggregates using Triton X-114 phase-partitioning to maximise E2-T1 yields. The suitability of this E2-T1 protein for small animal vaccination in mice is also demonstrated.

## Results

### Cloning and Expression

A truncated version of the E2 ORF frame was produced using PCR to remove the 3' region of the ORF which encodes for the membrane binding domain. The resultant 1040 bp PCR product was ligated into pET-SUMO expression vector.

Sequence analysis of pET-SUMO-E2-T1 demonstrated the vector encoded start codon, SUMO tag and 6-His region were in frame with E2-T1. The fusion protein encoded by pET-SUMO-E2-T1 has 463 residues; the amino acid composition is shown in Table 1. ProtParam was used to calculate the molecular weight and theoretical isoelectric point (pI) which were determined as 52.5 kDa and 6.22 respectively.

**Table 1:** Amino Acid composition of E2-T1

Amino acid	Number of residues	Amino acid	Number of Residues
Ala (A)	24	Leu (L)	32
Arg (R)	27	Lys (K)	31
Asn (N)	13	Met (M)	14
Asp (D)	33	Phe (F)	22
Cys (C)	17	Pro (P)	24
Gln (Q)	19	Ser (S)	23
Glu (E)	31	Thr (T)	30
Gly (G)	37	Trp (W)	6
His (H)	15	Tyr (Y)	16
Ile (I)	22	Val (V)	27

**Figure 1:**
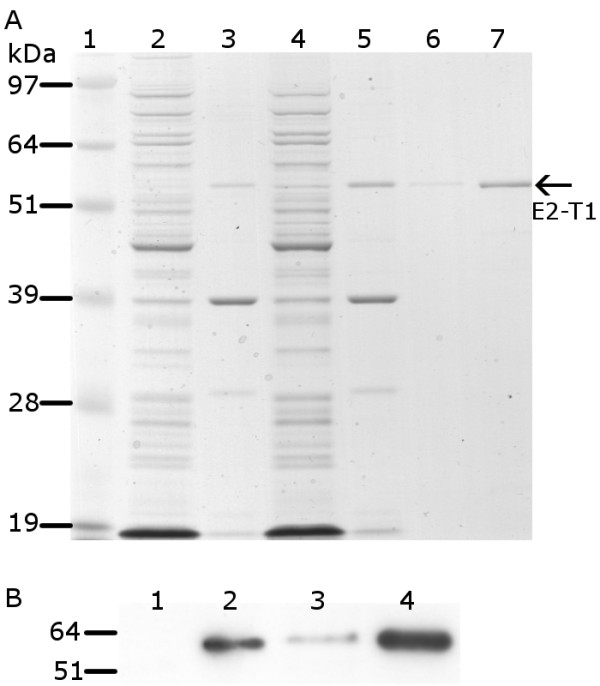
**Protein analysis of pET-SUMO-E2-T1 expression in *E. coli *BL21 (DE3)**. (A) Protein from soluble and insoluble fractions separated by electrophoresis on 10% Bis-Tris gel and stained with Coomassie blue. Lane 1, SeeBlue^® ^Plus2 MW standard; lane 2, soluble protein fraction at 0 hours; lane 3, insoluble protein fraction at 0 hours; lane 4, soluble protein fraction 2 hours post IPTG induction; lane 5, insoluble protein fraction 2 hours IPTG post induction; lane 6, E2-T1 purified from IB (0.4 μL); lane 7, E2-T1 purified from IB (2 μL). (B) Western blot image of E2-T1 protein expressed in *E. coli *BL21 (DE3). Equivalent amounts of protein from the soluble and insoluble fractions were transferred to Hybond C and incubated with an anti-his antibody and detected by ECL. Lane 1, soluble protein fraction 0 hours; lane 2, insoluble protein fraction 0 hours; lane 3, soluble protein fraction 2 hours post IPTG induction; lane 4, insoluble protein fraction 2 hours post IPTG induction.

Pilot expression studies indicated auto-induction of E2-T1 as insoluble protein at 0 hours. (Figure 1A, B). The optimal expression of E2-T1 was at 37°C, 2 hours post induction with 1 mM IPTG (Figure 1B). The majority of the E2-T1 protein was contained within the insoluble pellet fraction as inclusion bodies (Figure 1A).

### Purification and Refolding

Purification of E2-T1 from the soluble protein fraction was attempted using TALON resin. A step gradient of imidazole (50, 80, 100 and 150 mM) was used to determine the optimum elution profile. At 150 mM imidazole, E2-T1 was the major species eluted from the column. This eluted protein was of high purity but of low yield (10 ng/μL).

The majority of the E2-T1 protein was present in the insoluble fraction (Figure 1A, B). Using BugBuster™ it was possible to prepare soluble, high purity E2-T1 from the IB. E2-T1 protein was successfully dissolved in a DTT-SDS buffer (50 mM Tris (pH 6.8), 100 mM DTT, 1% SDS, 10% Glycerol). Titration of DTT concentrations using 5, 10 and 20 mM revealed a degree of solubility at all DTT concentrations; however, only at 100 mM DTT was the entire fraction of E2-T1 protein consistently solubilised (data not shown).

Refolding of E2-T1 protein was achieved by dialysis in 50 mM Tris (pH 7.0), 0.2% Igepal at room temperature. The resultant protein had high purity (Figure 1A, lane 6) with an average yield of 1-2 mg protein/litre of bacterial culture and was highly stable. E2-T1 stored at -20°C showed no degradation after more than 12 months and was resistant to degradation at room temperature for 7 days (data not shown).

**Figure 2:**
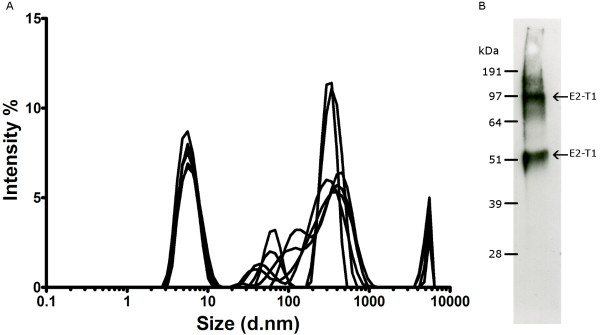
**(A) Dynamic Light Scattering analysis of E2-T1**. E2-T1 protein was dialysed into 50 mM Tris (pH 7.0) containing 0.2% Igepal CA630. The graph shows size distribution by intensity for physical size determination. Six measurements of 30 readings each were performed. (B) Western blot of purified E2-T1. E2-T1 protein was run under non-reducing conditions and probed with mouse sera raised against E2-T1.

Dynamic light scattering (DLS) analysis of purified E2-T1 demonstrated it to be a polydispersed protein with a polydispersity index (PdI) of 0.528 ± 0.041 indicating protein aggregation. The size distribution graph (Figure 2A) shows multiple species, a consistent major peak (37.5% ± 3.3% intensity) with a hydrodynamic diameter of 6.04 nm ± 1.65 nm is most likely a monomer species of E2-T1. This peak corresponds to a hypothetical globular protein of 44.7 ± 12.2 kDa as calculated by the Zetasizer software. The remaining peaks between 37 nm and 458 nm are representative of soluble protein aggregates. Peaks over 4,000 nm are most likely dust particles. A western blot of E2-T1 run under non-reducing conditions showed bands of 52 kDa and 100 kDa (Figure 2B). The 52 kDa band is consistent with the monomeric form as seen in the reducing gel (Figure 1B) and the 100 kDa suggests the formation of dimers of E2-T1.

Initial attempts to refold E2-T1 in 50 mM phosphate buffer (pH 6.0) and PBS (pH 7.2) were unsuccessful, as the protein formed insoluble aggregates, the addition of Igepal CA630 to 0.2% failed to improve protein solubility in these buffers. However, E2-T1 protein remained soluble in 50 mM Tris (pH 8.0), 0.2% Igepal CA630 and 50 mM Tris (pH 7.0), although in the absence of Igepal CA630, E2-T1 showed a much higher level of polydispersity (PdI: 0.931 ± 0.075).

### Endotoxin Removal

Endotoxin assays measured by Limulus Amebocyte Lysate (LAL) assay revealed levels ranging from 5.29 to 199.31 EU/mL (Table 2) for untreated batches of E2-T1.

**Table 2:** Purified protein concentrations and endotoxin levels

Sample ID	Protein Concentration ng/μL	Endotoxin Level EU/mL
1	200.80	110.32
2	279.15	199.31
4	265.32	29.91
5	92.50	5.29
6a*	76.92	< 3
6b*	111.34	< 3
7a*	74.16	< 3
7b*	138.88	< 3

* Samples treated with Triton X-114.

**Figure 3:**
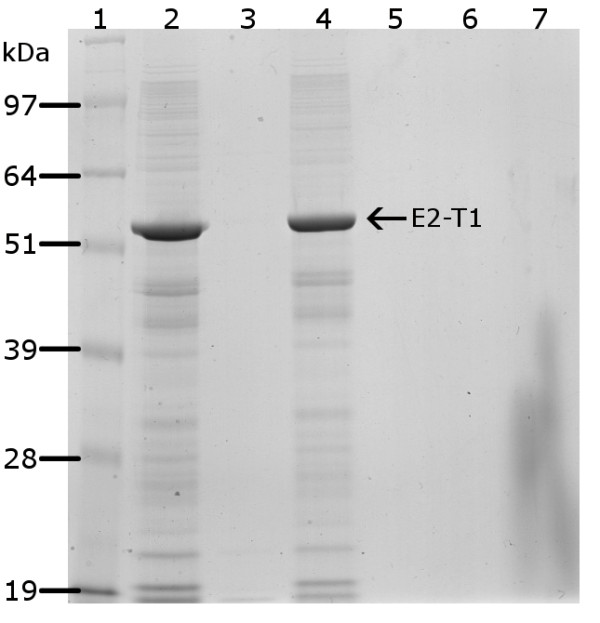
**Protein fractions following endotoxin removal from E2-T1 IB by Triton X-114 extraction were separated by electrophoresis on 10% Bis-Tris gel and stained with Coomassie blue**. Lane 1, SeeBlue^® ^Plus2 MW standards; lane 2, IB pellets resuspended in PBS before Triton X-114 treatment; lane 3, aqueous layer post Triton X-114 treatment; lane 4, recovered IB pellet post Triton X-114 treatment; lane 5, Triton TX-114 layer.

Treatment by phase separation using Triton X-114 on the insoluble IB pellets resulted in 3 phases: an aqueous phase, a detergent phase and the IB pellet fraction. Analysis of all three phases by SDS-PAGE showed that the protein was retained in the IB pellet with no loss of protein in the aqueous or oil phases (Figure 3). The resulting Triton X-114 treated E2-T1 samples had greatly reduced endotoxin levels below 3 EU/mL (Table 2). Due to the validation methods used for the LAL test, an absolute reading below this minimal threshold value could not be determined.

### Immunology

**Figure 4:**
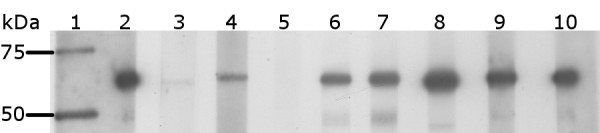
**Western blot analysis of E2-T1 protein with BVDV-specific antibodies**. Equivalent amounts of protein were transferred to Hybond C membrane and visualized by ECL. Lane 1, Precision Plus Protein Kaleidoscope MW standards; lane 2, Anti-His antibody; lane 3, sheep 804 pre immune sera; lane 4, sheep 804 post immune sera; lane 5, VMRD monoclonal D89; lane 6, VMRD monoclonal 157; lane 7, VMRD monoclonal 348; lane 8, Linfa Wang D4/G4 monoclonal; lane 9, VMRD Goat anti BVDV; lane 10, Anti-His antibody.

**Figure 5:**
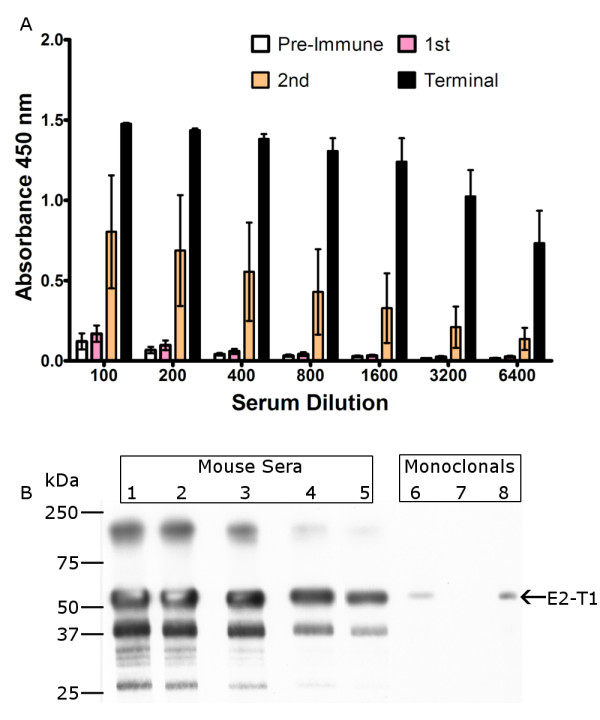
**Serum analysis from E2-T1 inoculated mice**. (A) ELISA analysis of mice receiving three injections of E2-T1. Mice (n = 4) were injected with 50 μg E2-T1 and 10 μg QuilA at 2 week intervals. ELISA assays were performed using pre-immune sera and sera obtained two weeks following each injection, termed 1^st^, 2^nd ^and terminal samples. (B) Western blot analysis of E2-T1 with terminal sera. Mice sera was used at the following dilutions: lane 1, 1:4000; lane 2, 1:8000; lane 3, 1:16000; lane 4, 1:32000; lane 5, 1:64000; lane 6, VMRD monoclonal 348; lane 7, VMRD monoclonal D89; lane 8, VMRD monoclonal 157.

Western hybridisation analyses showed solubilised E2-T1 protein was recognised by antibodies raised against BVDV in sheep and goats as well as 3 BVDV-E2 monoclonal antibodies, mAb-157, mAb-348 and mAb-9-D4 (Figure 4), the monoclonal mAb-89 failed to detect E2-T1 (Figure 4). To determine whether the bacterially expressed E2-T1 would be useful as a subunit vaccine candidate, mice were immunised with 50 μg purified E2-T1 in combination with 10 μg of QuilA subcutaneously in the tail base at 2 week intervals. The immune response was titrated by ELISA and Western blot analysis. The E2-specific antibody response by ELISA showed that following two injections the response is variable (yellow bars Figure 5A). However, after a third injection of E2-T1 protein an excellent humoral immune response in all 4 mice was detected (black bars Figure 5A).

The terminal bleed serum from one animal with the highest ELISA response was used in Western hybridisation analyses to E2-T1. Preimmune sera from this animal showed no reactivity at 1:200 and 1:4000 dilution (Data not shown). Specific detection of E2 protein was detected at high titre (1:64000) with mouse serum (Figure 5B). Non-specific bands were also observed, although E2-T1 was the dominant reactive species. The non-specific bands were not detectable by the preimmune sera.

## Discussion

The advantages of IB in terms of biological activity and facilitating protein purification have been highlighted in recent publications (15, 16). This study describes a procedure for the expression and solubilisation of a highly purified viral antigen derivative, E2-T1 from IB. The methodology includes the efficient removal of endotoxins to allow for potential *in vivo *applications.

*In vivo *refolding studies of purified E2 have shown that intramolecular disulphide bonds form rapidly within 2.5 minutes and intermolecular disulfide bonds result in formation of E2 dimers [7]. Since disulphide bonds are inefficiently formed in the reducing environment of the *E. coli *cytosol [8] and native E2 rapidly forms intramolecular disulphide bonds, misfolding of E2-T1 is likely to form an insoluble protein. Also, formation of intermolecular disulphide bonds between E2-T1 in the *E. coli *cytosol would lead to the formation of aggregates resulting in IB. In our study consistent and reproducible solubilisation of E2-T1 was only achievable using a highly reducing buffer containing 100 mM DTT. At lower DTT concentrations (20 to 5 mM), the percent solubility of E2-T1 decreased, indicating strong disulphide bond formation. The inclusion of DTT as a reducing agent in the re-suspension buffer would disrupt incorrectly formed disulphide bonds, and then during dialysis into DTT-free buffer, the disulphide bonds could reform in a conformation that is more suitable for soluble protein.

Although refolding of E2-T1 was successful in Tris based buffers, the resultant protein formed soluble aggregates as indicated by a PdI of 0.931. The soluble aggregation could be due in part to the same intermolecular disulphide bond formation reported for native E2. The addition of the detergent Igepal CA630 (0.2%) to Tris based buffers reduced aggregation (PdI 0.528), indicating some aggregation may be due to weaker intermolecular interactions such as ionic and/or hydrogen bonds. Under non-reducing electrophoresis conditions (Figure 2B), E2-T1 shows 2 dominant bands corresponding to monomeric and dimeric forms. This corresponds to the dimeric bonds between dimers arising from disulphide bond formation and other soluble aggregation is due to weaker intermolecular interactions. Addition of Igepal CA360 to PBS (pH 7.2) could not reverse the formation of aggregates indicating that the presence of Tris may be critical for maintaining E2-T1 solubility. The fact that a highly reducing buffer (100 mM DTT) was required to solubilise E2-T1 IB and the addition of Igepal CA360 could not completely overcome aggregation, lends evidence to strong disulphide bond formation.

Endotoxin levels lower then 3 EU/mL of *E. coli *derived proteins have been reported to be safe, causing no adverse reactions in animal trials [26,27]. However, in our case the E2-T1 protein purified from *E. coli *resulted in high endotoxin levels between 5.29 - 199.31 EU/mL. This was considered unsuitable for testing E2-T1 as a component of a veterinary vaccine due to the risk of adverse immunological reactions. Commercially available techniques for endotoxin removal typically employ affinity columns and require buffer changes for washing and elution of the protein. Attempts to use ion exchange chromatography were discontinued due to consistently low recovery of E2-T1.

We introduced a significantly innovative step for endotoxin removal from the *E. coli *IB by adding Triton X-114 in the E2-T1 expression and purification protocol prior to solubilisation and recovery of the target protein. Phase separation with Triton X-114 resulted in up to 600 fold reduction in the endotoxin levels of the E2-T1 preparations (Table 2). Importantly, using this method resulted in no detectable E2-T1 loss (Figure 3), as the protein remained insoluble within the IB. This approach could be generally applicable for the efficient removal of endotoxins from other proteins contained within IB preparations, by maintaining the IB in a buffer incompatible with the solubilisation of the target protein, while still facilitating endotoxin removal with Triton X-114.

We have shown that E2-T1 protein produced in *E. coli *was recognised by antibodies raised in goat and sheep against whole BVDV and also by various BVDV-specific monoclonal antibodies (Figure 4) indicating the usefulness of E2-T1 for diagnostic applications such as monitoring the serological status of animals which are the natural host of BVDV. Furthermore an ELISA done using E2-T1 against 5 BVDV infected cattle sera samples with known serum neutralisation titres showed that E2-T1 is recognised by cattle sera at a comparable level to the commercial positive control sera (data not shown).

One monoclonal antibody, mAb-D89, did not recognise E2-T1, which could be due to its binding to either a conformational E2 epitope or an epitope that is dependent on post-translational modification of E2 such as glycosylation. As the binding capacity of mAb-D89 was only assessed under denaturing and reducing conditions this could have caused the loss of the mAb-D89 epitope from the renatured E2-T1. It remains to be determined how the potential loss of spatial and other non-linear epitopes of native E2 would affect the efficaciousness of any vaccine formulations containing E2-T1.

Immunisation of mice with bacterially derived E2-T1 protein resulted in an excellent immune response, confirmed both by ELISA assay and Western blot hybridisation. Western blot analysis with mouse sera collected after three immunisations with E2-T1 resulted in very strong detection of E2 protein with significant bands readily detectable at a 1:64000 dilution. The predominant lower molecular weight band (approximately 45 kDa) observed by Western hybridisation (Figure 5B) may be due to the polyclonal nature of the antibodies and are not present when probed with pre-immune sera.

The results of this study demonstrate that the bacterially derived E2-T1 protein solubilised from IB can induce an excellent humoral response in small animals and could potentially be used as a subunit vaccine. The next step will be to determine if E2-T1 can elicite similar responses in large animals such as sheep or cattle when evaluated as a potential vaccine antigen.

## Conclusion

The development of cost effective veterinary vaccines and diagnostics requires the use of efficient methods that enable the production of large quantities of immunogenic and non-toxic proteins. Using an *E. coli *system and exploiting the formation of IB, we were able to produce a highly pure, stable and endotoxin-free E2-T1 protein. It was highly immunogenic in mice and was recognised by BVDV-E2 specific antibodies raised to native E2 in BVDV. We have developed a procedure for solubilising and refolding E2-T1 as well as the removal of endotoxin by Triton X-114 two-phase extraction which offers an elegant solution for the production of other proteins intended for diagnostic uses and *in vivo *uses such as subunit vaccines or therapeutics.

## Methods

### Cloning of E2-T1 into pET-SUMO bacterial expression vector

The E2 gene was amplified from a plasmid containing BVDV isolate MD74 which has been identified as a type-1 isolate [28]. Twenty μL of Qiagen (Venlo, The Netherlands) PCR master mix was used with primers at a final concentration of 0.5 μM. The forward primer nanoE2-F sequence was 5'-ATGGTGGATCCGTGCAAGCCT-3' and the reverse primer nanoE2-Rtrunc1 sequence was 5'-CTAAGACTCGGCGAAGTAGTCCCGG-3'. PCR cycling conditions comprised an initial incubation at 95°C for 5 minutes, followed by 35 cycles at 94°C for 30 seconds, 60°C for 30 seconds and 72°C for 90 seconds. The resultant 1040 bp product was ligated into the pET-SUMO vector (Invitrogen, Carlsbad, USA). The ligation products were subsequently transformed into electrocompetent *E. coli *strain DH10B (Invitrogen). Positive clones were confirmed by sequencing (AGRF, Brisbane, Australia) and transformed into *E. coli *strain BL21 (DE3, Invitrogen) cells for protein expression.

### Large-scale expression and purification of E2-trunc1 protein

An overnight culture of *E. coli *BL21 (DE3) containing pET-SUMO-E2-T1 was used to inoculate four 250 mL cultures of LB Miller broth (Amresco, Solon, USA) containing 50 mg/L Kanamycin-sulphate (Amresco). These cultures were grown at 37°C to an OD_600 _of 0.4 to 0.6, then induced with 1 mM IPTG and grown for a further 2 hours. The bacterial pellet was collected by centrifugation at 3,800 *g*, at 4°C for 15 minutes in 4 × 250 mL centrifuge tubes. Total protein was extracted by resuspending each bacterial pellet in 50 mL *E. coli *lysis buffer (50 mM KPO_4 _phosphate (pH 7.8), 400 mM NaCl, 100 mM KCl, 10% glycerol, 0.5% Triton X-100, 10 mM Imidazole), with the addition of 12.5 mg Lysozyme and 750 units of Benzonase nuclease (Novagen-Merck, Darmstadt, Germany). The bacterial suspensions were incubated in lysis buffer for 20 minutes with gentle shaking. The samples were frozen in liquid nitrogen, and thawed at 42°C three times. The resultant solution was centrifuged at 37,000 *g *at 4°C for 15 minutes. The insoluble protein fraction (containing IB aggregates) and the supernatant (containing the soluble protein fraction) were stored at -20°C until required.

### Purification of soluble E2-T1

E2-T1 protein was purified from the soluble fraction by affinity chromatography using TALON (Clontech, Mountain View, USA) resin following manufacturer's instruction. Bound protein was eluted from the resin using TALON elution buffer with a step gradient of 50 mM, 80 mM, 100 mM and 150 mM imidazole, collecting ten 500 μL fractions/step. Fractions were analysed by SDS-PAGE.

### Purification of inclusion body aggregates of E2-T1

The insoluble protein fraction (including E2-T1) was recovered from the IB using BugBuster™ Master Mix (Novagen-Merck) as follows. IB pellets (equivalent to 160 mL bacterial culture) were resuspended in 2.5 mL BugBuster™ and vortexed for 2 minutes. Following the addition of 15 mL of 1:10 diluted BugBuster™ and further vortexing for 1 minute, the resuspended IB pellets were centrifuged at 5000 *g *at 4°C for 15 minutes. Three further washes of 25 mL 1:10 diluted BugBuster™ were performed with vortexing and centrifugation steps as above. Following the final wash step, the pellets were resuspended in 1 mL 1:10 diluted BugBuster™, transferred into 1.5 mL tubes and centrifuged at 16,200 *g *at 4°C for 15 minutes. The supernatant was removed and the IB pellets were stored at -20°C without any detectable protein degradation.

### Endotoxin Removal

All reagents were prepared in endotoxin-free water (< 0.001 EU/mL, MO BIO Laboratories, Carlsbad, USA). Triton X-114 exhibits a cloud point at 22°C, above this temperature micelles aggregate forming a new phase with very low water content. Endotoxin remains in the detergent phase [21]. Purified IB pellets were resuspended in 1 mL of PBS (137 mM NaCl, 2.7 mM KCl, 10 mM Phosphate buffer (pH 7.2), Amresco), and vortexed for 1 minute to disperse the insoluble protein. Dispersed protein solutions were mixed with 1% (v/v) of Triton X-114 by vigorous vortexing for 1 minute. Samples were incubated on ice for 5 minutes, vortexed and subsequently incubated at 56°C for 1 minute to allow phase separation. After centrifugation at 16,200 *g *at room temperature for 7 seconds in a microfuge the 3 phases (aqueous, oil and pellet) were recovered into separate tubes and analysed by SDS-PAGE electrophoresis.

To determine the level of endotoxin in the protein samples, endotoxin assays were performed by using the Limulus Amebocyte Lysate (LAL) assay by AMS Laboratories (Sydney, Australia).

### Solubilisation of E2-T1

Protein pellets from the IB preparations were dissolved in 50 mM Tris (pH 6.8), 100 mM DTT, 1% SDS, 10% Glycerol, vortexed at low speed for 2 minutes and incubated at 37°C for 20 minutes. The resulting solubilised protein was dialysed at room temperature, with 3 buffer changes over 24 hours against 50 mM Tris (pH 7.0), 0.2% Igepal CA630 (Sigma-Aldrich). Following dialysis protein integrity was determined by SDS-PAGE analysis and protein yield determined by colourimetric assay (BioRad DC Kit, Hercules, USA). Other dialysis buffers evaluated in this study were: 50 mM Tris, (pH 7.0); 50 mM Tris (pH 8.0), 0.2% Igepal CA630; PBS (pH 7.2); PBS (pH 7.2), Igepal CA630; 50 mM phosphate buffer (pH 6.0).

### SDS-PAGE Electrophoresis

SDS-PAGE analysis was performed using Invitrogen's XCell *SureLock*^® ^Mini-Cell precast system with NuPAGE 10% BIS-Tris gels according to manufacturer instructions. Size estimations were determined against SeeBlue^® ^Plus2 (Invitrogen) or Precision Plus Protein Kaleidoscope Standards (BioRad) pre-stained standards. The resolved proteins were visualised by staining in 50% methanol, 10% acetic acid, 0.25% Coomassie Blue R250 for 30 minutes, followed by destaining in 30% methanol, 10% acetic acid for 10 minutes three times.

### Western Hybridisation Analysis

Following SDS-PAGE electrophoresis the resolved polypeptides were transferred to Hybond C nitrocellulose membrane (GE Healthcare, Buckinghamshire, United Kingdom) using Invitrogen XCell II™ Blot Module Kit according to manufacturer's instructions. All antibodies were diluted in BLOTTO (PBS (Amresco), 0.1% Tween 20, 1% skim milk). E2 specific monoclonal antibodies D89 [29], mAb-157 and mAb-348 [30] and polyclonal goat anti-BVDV (VMRD, Pullman, USA). The pestivirus genus specific monoclonal 4-9D4 was kindly provided by Dr Linfa Wang [31]. E2 specific monoclonal antibodies were used at 1:100 dilution. An anti-E2 sheep sera 804 was produced in vaccinated sheep after intramuscular injection of BVDV E2 and diluted to 1:500. Monoclonal 6× His antibodies (Clontech) were used at 1:15,000. Anti mouse immunoglobulin G HRP conjugate (Chemicon, Millipore, Billerica, Massachusetts, USA) and anti sheep/goat immunoglobulin HRP conjugate (Sigma) were used at 1:2,000 and 1:10,000 respectively. Detection was carried out using an ECL detection kit (GE Healthcare).

### Animals

C57BL/6J mice were purchased from and housed in the Biological Resource Facility, The University of Queensland, Brisbane, Australia under specific pathogen free conditions. Female mice were housed in HEPA-filtered cages with 4 animals per cage in an environmentally controlled area with a cycle of 12 hours of light and 12 hours of darkness. Food and water were given *ad libitum*. All studies were conducted with 8 week old mice at the time of first injection. All procedures were approved by The University of Queensland Ethics Committee.

### Immunisation of mice with E2-T1

Pre-immunisation blood samples of were collected by retro-orbital bleed using heparin coated hematocrit tubes (Hirschmann Laborgeräte Heilbronn, Germany). Pre-immunisation blood samples collected prior to the first immunisation were referred to as the preimmune samples. Blood samples collected 2 weeks after the third immunisation was referred to as terminal bleed.

All doses of E2-T1 were prepared in sterile conditions in a certified biological safety cabinet using sterile reagents, equipment and aseptic technique. The adjuvant QuilA (Superfos Biosector, Vedback, Denmark) was resuspended at 2 mg/mL in sterile injectable water (Pfizer, Brooklyn, USA).

Dose volumes were adjusted to 500 μL using 0.9% saline (Pfizer). 50 μg E2-T1 together with 10 μg QuilA, were administered in a final volume of 100 μL subcutaneously at the tail base using a sterile 27 gauge needle (Terumo, Tokyo, Japan). Three injections were administered at 2 week intervals and mice were sacrificed 14 days after the final immunisation. Animals were closely monitored throughout the study. All the animals remained in good health for the duration of the study with no visible deleterious health effects.

### Humoral antibody responses

ELISA assays for the detection of E2-T1-specific antibodies were performed by coating microtitre plates (96 well, Nunc, Maxisorb, Roskilde, Denmark) with

50 μL E2-T1 antigen solution (2 ng/μL in PBS) overnight at room temperature. Antigen solution was removed from the plates which were then washed once with 1× PBS-T (1× PBS, 0.1% Tween: Sigma-Aldrich) and blocked with 200 μL per well of PBS containing 5% BSA (Sigma-Aldrich), 5% skim milk (Fonterra, Auckland, New Zealand) for 1 hour with gentle shaking at room temperature. Plates were washed three times in 200 μL 1× PBS-T as described above.

Mouse sera samples were diluted from 1:100 to 1:6400 and 50 μL of diluted sera was added to plates then incubated for 2 hours at room temperature. To detect mouse antibodies HRP conjugated polyclonal sheep anti-mouse IgG antibodies (100 μL of 1:10000 dilution in PBS (pH 7.2), Chemicon Australia, Melbourne, Vic, Australia) was added per well and incubated for 1 hour at RT with gentle shaking. Plates were washed three times in 200 μL 1× PBS-T and 100 μL of TMB substrate (Sigma-Aldrich) was added to each well. After 15 minutes at RT 100 μL of 1 N HCl was added to each well to stop the chromogenic reaction. Plates were read at 450 nm within 10 minutes.

### Concentrating of E2-T1/Buffer Change

Protein concentration was increased by using 30 kDa Vivaspin 2 centrifugal filters (Millipore). Briefly, up to 2 mL of protein solution was added to the upper chamber and centrifuged (4000 *g*, 4°C) until the volume was reduced to the required amount. These columns were also used to perform buffer changes. After concentration of samples, the target buffer was replenished to the original volume.

### Dynamic light scattering analysis

Size and polydispersity data were determined by dynamic light scattering (DLS) using a Malvern Instruments Zetasizer Nano (Worcestershire, UK.). E2-T1 solution (1 mL) was placed in a clear disposable zeta cell (DTS1060C, Malvern Instruments). Analysis was performed using the size standard operating procedure (SOP) for which the following parameters were use: material set to protein, dispersant set to water, taking 5 - 6 independent measurements of 30 runs each. The size and polydispersity index for samples were the average of all measurements. Analysis of the data was carried out by Malvern Zetasizer software.

### Physical properties of E2-T1

Physical data was obtained using ProtParam, last accessed 3 March 2011 at http://au.expasy.org/tools/protparam.html.

## Competing interests

The authors declare that they have no competing interests.

## Authors' contributions

ASC and DM carried out all experimental work excluding the Western hybridisations. MC performed the western hybridisations. TJM and NM supervised the study and participated in its design and coordination. ASC, DM, TJM and NM were all involved in the experimental design. All authors read and approved the final manuscript.
